# Diagnostic Challenges in Childhood Pulmonary Tuberculosis—Optimizing the Clinical Approach

**DOI:** 10.3390/pathogens11040382

**Published:** 2022-03-23

**Authors:** Kenneth S. Gunasekera, Bryan Vonasek, Jacquie Oliwa, Rina Triasih, Christina Lancioni, Stephen M. Graham, James A. Seddon, Ben J. Marais

**Affiliations:** 1 Department of Epidemiology of Microbial Diseases, Yale School of Public Health, New Haven, CT 06510, USA; 2 Department of Pediatrics, University of Wisconsin School of Medicine and Public Health, Madison, WI 53726, USA; vonasek@wisc.edu; 3 Health Services Unit, KEMRI-Wellcome Trust Research Programme, Nairobi P.O. Box 43640-00100, Kenya; joliwa@kemri-wellcome.org; 4 Department of Paediatrics and Child Health, School of Medicine, University of Nairobi, Nairobi P.O. Box 30197-00100, Kenya; 5 Department of Pediatrics, Faculty of Medicine, Public Health and Nursing, Universitas Gadjah Mada/Dr. Sardjito Hospital, Yogyakarta 55284, Indonesia; rina_triasih@yahoo.com; 6 Department of Pediatrics, School of Medicine, Oregon Health and Science University, Portland, OR 97239, USA; lancioni@ohsu.edu; 7 Centre for International Child Health, University of Melbourne and Murdoch Children’s Research Institute, Royal Children’s Hospital, Melbourne, VIC 3052, Australia; steve.graham@rch.org.au; 8 Burnet Institute, Melbourne, VIC 3004, Australia; 9 Department of Infectious Diseases, Imperial College London, London W2 1PG, UK; james.seddon@imperial.ac.uk; 10 Desmond Tutu TB Centre, Department of Paediatrics and Child Health, Stellenbosch University, Cape Town 8000, South Africa; 11 University of Sydney and The Children’s Hospital at Westmead, Sydney, NSW 2145, Australia; ben.marais@health.nsw.gov.au

**Keywords:** tuberculosis, children, paediatric, diagnosis, symptom-based

## Abstract

The management of childhood tuberculosis (TB) is hampered by the low sensitivity and limited accessibility of microbiological testing. Optimizing clinical approaches is therefore critical to close the persistent gaps in TB case detection and prevention necessary to realize the child mortality targets of the End TB Strategy. In this review, we provide practical guidance summarizing the evidence and guidelines describing the use of symptoms and signs in decision making for children being evaluated for either TB preventive treatment (TPT) or TB disease treatment in high-TB incidence settings. Among at-risk children being evaluated for TPT, a symptom screen may be used to differentiate children who require further investigation for TB disease before receiving TPT. For symptomatic children being investigated for TB disease, an algorithmic approach can inform which children should receive TB treatment, even in the absence of imaging or microbiological confirmation. Though clinical approaches have limitations in accuracy, they are readily available and can provide valuable guidance for decision making in resource-limited settings to increase treatment access. We discuss the trade-offs in using them to make TB treatment decisions.

## 1. Background

Despite growing awareness that tuberculosis (TB) is a major preventable cause of death among children, most eligible children are not provided with TB preventive treatment (TPT) and many children die due to limited or delayed access to treatment for TB disease [1]. A major factor limiting treatment access is the absence of diagnostic approaches that are feasible and scalable in resource-limited settings across the full spectrum of TB disease and health services delivery. The urgent need to improve TB prevention and care among children, especially those who are young and vulnerable, has been highlighted in the “Roadmap towards Ending TB in Children and Adolescents” launched at the United Nations (UN) High-Level meeting on Ending TB in 2018 [2]. However, progress towards the targets articulated in the Roadmap and endorsed by member states at the UN High-Level meeting remains limited [3].

In this article we focus on clinical approaches to guide TB treatment and prevention among children under 10 years of age who develop a different disease spectrum compared to adolescent and adult patients [4]. TB among these younger children tends to be paucibacillary, resulting in a reduced microbiological diagnostic yield [5]. In addition, the collection of respiratory specimens from children who are unable to expectorate is challenging [6]. Diagnostic approaches that carefully consider clinical signs and symptoms may provide a feasible strategy to reduce TB mortality in children.

The aim of this article is to provide a brief overview and practical guidance to healthcare workers on clinical approaches to make treatment decisions for children by synthesizing the latest evidence and guidelines. We will consider two applications of clinical approaches: (1) use as a “rule-out” test in screening to guide the initiation of TPT in close contacts of infectious TB cases and children living with HIV; and (2) use in TB treatment decision making for children presenting to healthcare with symptoms and signs suggestive of TB.

### 1.1. Symptom-Based TB “Rule-Out” to Guide TPT Use

In high-TB incidence settings with resource limitations, symptom-based screening is an effective, safe, and practical strategy for assessing children at high risk for TB. Here, we focus on two high-risk groups: (1) children recently exposed to an infectious case of TB and (2) children living with HIV. Symptom-based screening can be used to differentiate between (1) children who screen negative (less likely to have TB disease) and may benefit from TPT and (2) children who screen positive (more likely to have TB disease) and need further investigation to initiate TB treatment.

#### 1.1.1. Summary of the Evidence: TB Contacts

Recent household exposure identifies a high-risk event, especially for vulnerable young children (<5 years) [7]. Screening for TB through household contact investigation presents an opportunity for early detection and treatment of prevalent TB and prevention of disease through TPT. Most children who develop TB do so within 12 months following primary infection [7]. Those without prevalent TB at the time of screening are within this high-risk window for disease progression, making TPT a highly effective prevention strategy [8]. In order to maximize delivery of TPT among young household contacts in resource-limited settings, a simple approach with a high negative predictive value for TB is needed.

A Cochrane Review of screening tests for TB in children included a meta-analysis of the accuracy of symptom-based screening among four studies of child TB contacts [9]. The pooled sensitivity of symptom-screening for TB was 89% (95% confidence interval (CI): 52% to 98%) and the pooled specificity was 69% (95% CI: 51% to 83%). Variation in the symptom definitions used and the specifics of the screening process limited study comparability. Two of the studies included in this meta-analysis also provide data on chest X-ray (CXR) findings in asymptomatic child TB contacts [10,11], which we explore in greater detail.

In a prospective cohort study of child TB contacts aged ≤15 years and followed for 12 months in Yogyakarta, Indonesia [10], a positive symptom screen was defined as any of: current cough, fever, poor appetite, weight loss, failure to thrive, hemoptysis, fatigue, or night sweats. All children recruited into the study underwent a comprehensive investigation, including tuberculin skin testing (TST) and CXR. Of 269 children recruited, 21 were found to have prevalent TB. Table 1 shows the sensitivity, specificity, and negative predictive value calculated for the symptom-based screening for TB in this study. In a prospective observational study conducted in Cape Town, South Africa [11], study nurses screened 252 child TB contacts <5 years for any current TB symptoms: fever, cough, wheeze, reduced playfulness/unusual fatigue, or failure to thrive/ weight loss. This study utilized different reference standards to determine accuracy estimates for symptom-based TB screening, as shown in Table 1.

CXR is routinely used in well-resourced settings to evaluate TB contacts; however, CXR is rarely available in resource-limited settings in which the majority of TB cases are found. Fortunately, available data suggests that asymptomatic young child contacts may be initiated on TPT without CXR. Uncomplicated hilar lymphadenopathy is a common finding on CXR following recent primary infection in young children [4,12], and is sometimes treated as TB; however, only a small percentage of these children will develop symptomatic TB. In the South African study mentioned above [11], among 175 children with a negative symptom screen, 8 children had uncomplicated hilar adenopathy on CXR and were treated for TB. In the Indonesian study [10], of children with a negative symptom screen, 11 (13%) were found to have hilar lymphadenopathy. None received TB treatment and none developed TB disease during the 12-month follow-up period.

**Figure 1:**
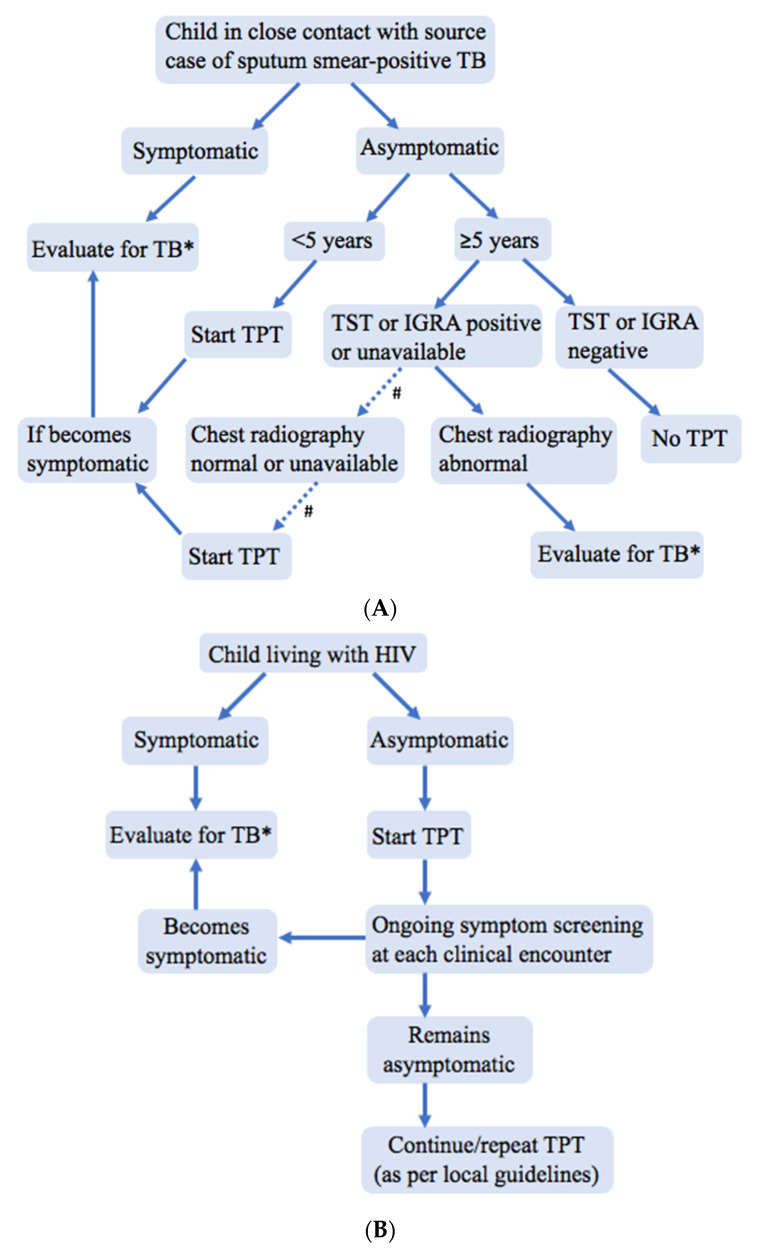
Suggested algorithm to manage (**A**) HIV-negative child tuberculosis contacts and (**B**) children living with HIV when chest X-rays and tests of infection are not readily available; adapted from and consistent with World Health Organization guidance [14]. CXR—chest X-ray, IGRA—interferon-gamma release assay, TB—tuberculosis, TPT—TB preventive treatment, TST—tuberculin skin test. # Evidence is limited regarding the benefits and risks of TPT in asymptomatic child TB contacts ≥5 years of age without a TST or IGRA to document infection and without a CXR or other sensitive test to rule out TB disease and among children ≥10 years for whom higher bacillary load is disease is more common. * If evaluation definitively rules out TB disease, then TPT should be started.

The World Health Organization (WHO) now also recommends TPT for older child (5–9 years) and adolescent (10–19 years) TB contacts. The evidence and programmatic experiences informing the use of CXR in these older groups is limited. However, since bacteriologically positive pulmonary TB is common in adolescents and adults not reporting symptoms [13], WHO guidelines advise a CXR for older TB contacts before commencing TPT due to concerns that subclinical TB may not be appropriately treated (Figure 1A) [14].

#### 1.1.2. Summary of the Evidence: Children Living with HIV in High-TB Incidence Settings 

For children living with HIV, WHO recommends a similar symptom-based screening approach to TPT provision as is recommended for young child TB contacts. Any current cough, fever, or poor weight gain constitutes a positive symptom screen, requiring further investigation for TB, as does a history of TB exposure. Children aged 1 year or older living with HIV in high-TB incidence settings should receive TPT if there are no contraindications and they have a negative symptom screen, regardless of whether the child is receiving anti-retroviral therapy or has documented TB exposure [14]. In addition, ongoing symptom screening at each clinical encounter is needed to assess for future TB disease, which remains a risk irrespective of previous TPT. CXR is not necessary before commencing TPT in asymptomatic children living with HIV, including those 5 years of age and older (Figure 1B).

A prospective study of the WHO symptom-based screening approach among 247 South African HIV-positive children aged <8 years reported 57% sensitivity and 97% specificity to identify TB disease [15]. A larger retrospective study of this screening approach among 20,706 HIV-positive patients aged <19 years in six African countries reported similar performance: 61% sensitivity and 97% specificity [16]. These low sensitivities are concerning given the potential for rapid progression of TB disease and death among children living with HIV [17]. The high specificities reported are counterintuitive given how ubiquitous these symptoms are among HIV-positive children. More research is needed in this area, but these results emphasize the importance of close clinical follow-up, including repeated symptom screening for TB at every clinical encounter, to improve sensitivity.

#### 1.1.3. Recommended Approach in Resource-Limited Settings

The reality in most TB-endemic areas is that clinics cannot effectively screen and treat child TB contacts and HIV-positive children unless pragmatic approaches are adopted that take account of available resources. Implementing a simple symptom-based approach makes screening more feasible (Table 2) and should improve TPT access.

The available evidence supports current WHO recommendations for a symptom-based approach to screening of child TB contacts <5 years and of HIV-positive children (Figure 1) living in TB-endemic areas with limited resources [14]. This approach aims to improve TPT access for those at greatest risk of TB disease and death. For child TB contacts, one-off screening can be utilized to determine eligibility for TPT, and screening should be repeated if there is future re-exposure. For children living with HIV, screening is performed for the initial determination of TPT eligibility, but screening for TB disease and repeat TB exposure should be ongoing at each clinical encounter. 

### 1.2. Symptom-Based Approach to Guide TB Treatment Use

WHO guidance suggests that children brought to healthcare services with suggestive TB symptoms (a presumptive TB case) should be further evaluated for TB disease [18]. Once a child has been identified as a presumptive case, healthcare workers must consider whether to initiate TB treatment based upon the clinical history, physical examination, demographic data, history of recent exposure to a TB source case in the preceding 12 months, confirmatory tests for *M. tuberculosis*, chest imaging, tests of infection, and clinical follow-up where appropriate. Treatment decisions must often be made in the absence of microbiological confirmation. Thus, symptoms, clinical examination, and history of close TB contact play a crucial role in the decision to initiate TB treatment.

The evidence supporting the role of symptom-based diagnosis to inform TB treatment decisions has been limited due to poorly standardized symptom and case definitions, few validation studies, and challenges in designing studies that adequately evaluate the role of individual symptoms and variable symptom combinations. In the following section, we discuss an overview of existing clinical approaches and the trade-offs in making TB treatment decisions. Finally, we describe new WHO guidance that standardizes clinical approaches to support rapid and uniform treatment decision making for presumptive TB cases [19].

#### 1.2.1. Overview of Existing Approaches/Evidence

A detailed clinical history and physical examination may be the only evidence available to inform TB treatment decisions [6]. While a presumptive case may be defined as a child with one or more symptoms or signs suggestive of TB, whether the child should receive TB treatment requires careful consideration of all relevant information, including the duration and character of the symptoms and signs, recent TB exposure, and the results of available tests. 

In most children, pulmonary TB presents with subacute symptoms, which explains the emphasis on symptoms of a longer duration. The character of the cough may provide additional information of value. A persistent, non-remitting cough, especially if associated with weight loss or failure to thrive, was found to be strongly associated with pulmonary TB, while intermittent cough or wheeze were associated with other diagnoses [20]. However, in very young children (< 2 years of age) TB may present with more acute respiratory symptoms, which explains why “any current cough” is used for contact screening and why TB should be considered in the differential diagnosis of young children with acute symptoms, especially if recent exposure is reported. “Failure to thrive” may also convey different degrees of certainty; for example, objective evidence of weight loss or crossing of percentile lines on standard growth curves carries more certainty than caregiver-reported, subjective history of weight loss. To aid decision making, weight and height should be plotted on standard growth curves at each clinical visit whenever possible. Weight is also important to guide treatment dosage decisions and to monitor treatment response.

It is critical to ask whether the child may have been exposed to a microbiologically confirmed case of pulmonary TB within the previous 12 months. If so, it is reasonable to assume a higher likelihood for TB [7]. If a known contact is not reported, further questioning may identify contact with someone who has suggestive symptoms and as yet undetected or untreated disease. If the likely source case had drug-resistant TB, then their drug susceptibility test profile should guide the treatment, including preventive treatment, of close contacts as well. 

Physical evaluation may detect signs suggestive of pulmonary TB, but it is rarely informative and generally more helpful to guide alternate diagnoses. Abnormal vital signs or breathing difficulty (e.g., chest-wall retractions, paradoxical breathing, use of intercostal muscles) may indicate an acute condition that requires immediate management. In general, pulmonary TB in children presents with minimal signs or symptoms suggestive of acute disease. In fact, a perceived discrepancy between the severity of clinical and radiological disease is an important pointer to consider TB.

Children are at increased risk for extrapulmonary TB (EPTB) as compared to adults [21]. EPTB manifests heterogeneously with various symptoms and signs, the most concerning of which is TB meningitis. Children who present with lethargy, signs of raised intra-cranial pressure, reduced consciousness, focal neurological deficits, and/or unexplained seizures in a TB-endemic setting should raise concern for TB meningitis. TB meningitis can be challenging to diagnose and prompt empiric treatment is often required to reduce morbidity and mortality; early diagnosis and treatment are critically important [22]. The most common EPTB manifestation is cervical lymph adenitis. In high-TB incidence settings, the presence of a large cervical lymph node mass (>2 × 2cm) that is matted and non-tender is highly suggestive of TB lymph node disease [23,24].

#### 1.2.2. Important Trade-Offs in Deciding to Initiate TB Treatment

Many children presenting to healthcare services in high-TB incidence settings have symptoms that could be suggestive of TB, such as malnutrition, cough, and fever. Furthermore, the features associated with TB described in the section above are not unique to TB and may overlap with other diseases. Comprehensive TB investigation is complicated by potential delays or an inability to perform high quality CXR, or microbiological testing for *M. tuberculosis*. Negative results for microbiological testing do not rule out TB due to limitations in sensitivity, and smear microscopy of respiratory specimens has limited diagnostic value in young children [25]. Positive results for tests of *M. tuberculosis* infection (i.e., tuberculin skin test or interferon-gamma release assays) may increase the likelihood of TB in the presence of symptoms but has limited value in areas where TB infection is common. A more comprehensive review of laboratory-based diagnostics for childhood TB is provided by Marcy et.al. in this series (unpublished at the time of this submission).

Within this context, healthcare workers in resource-limited settings are often left to make a TB treatment decision based on limited information. There options are to start TB treatment at the initial visit or to withhold/delay treatment (either to await results from imaging/microbiological testing or to re-evaluate the child at follow-up). Both decisions have to consider important clinical trade-offs (Table 3).

A major consequence of withholding/delaying treatment is the risk of rapid progression of disease, which is more common in groups at higher risk of TB-associated mortality, including children living with HIV who are severely immunocompromised, very young children (<2 years), and children with severe acute malnutrition. Given the increased risk of rapid disease progression and mortality in these risk groups, healthcare workers may lower the threshold for treatment and consider making same-day treatment decisions.

With immediate TB treatment there is a risk of missing alternate diagnoses and potentially exposing the child to drug-related adverse events associated with unnecessary TB treatment. The implications of missing alternate diagnoses vary by setting. For example, children presenting with symptoms suggestive of TB may have common lower respiratory tract infections requiring a short course of antibiotics or no treatment at all, while children presenting with fever may require a course of antimalarial treatment. Treating for alternate non-TB diagnoses and then reevaluating the child would reduce unnecessary TB treatment and the consequences of missed or delayed treatment of alternate diagnoses. This is especially reasonable among children at lower risk of TB disease progression. If treatment of an alternate diagnosis is initiated, clinical follow-up to ensure resolution of presenting signs and symptoms is critical to ensure that underlying TB disease is not missed.

#### 1.2.3. Proposed New Approach Using Treatment Dcision Algorithms

The high mortality associated with untreated childhood TB requires practical guidance to identify and treat more children with TB using the best available data. Treatment decision algorithms and scoring systems provide structures to evaluate and promote rapid and uniform treatment decision making by assigning scores to evidence and/or decision points to guide evaluations [26,27,28]. Recent approaches to algorithm building have used data from the best available diagnostic studies to specify which features might be sufficient to begin treatment in the absence of microbiological confirmation of *M. tuberculosis* [29,30].

**Figure 2:**
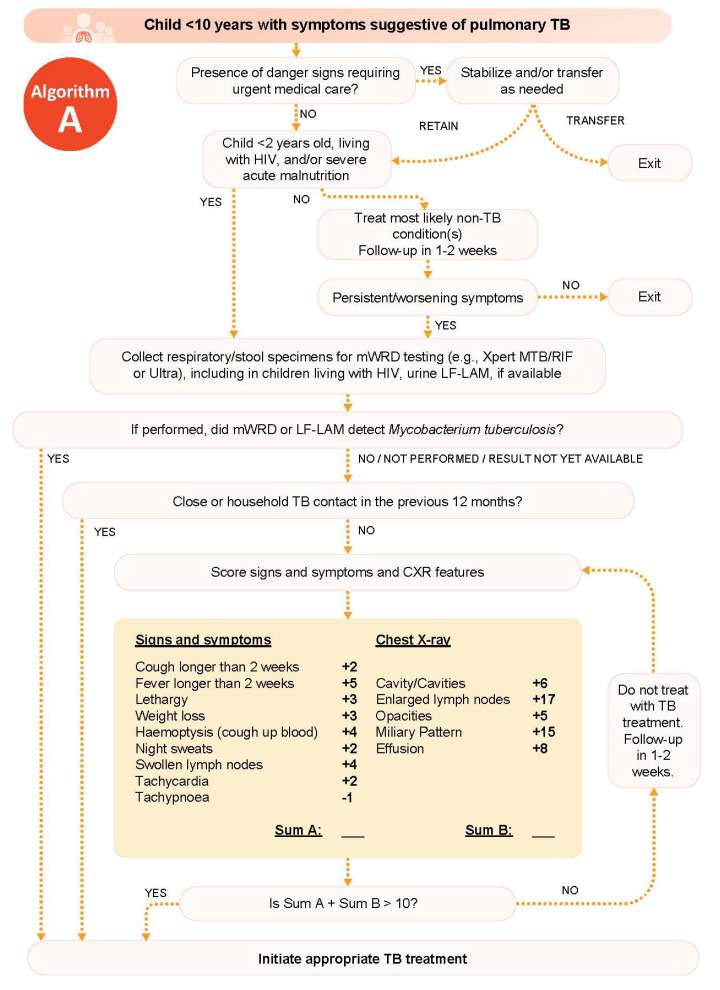
Tuberculosis treatment decision algorithm in children less than 10 years of age with symptoms suggestive of pulmonary tuberculosis, reproduced from the operational handbook accompanying the 2022 consolidated guidelines on the management of TB in children and adolescents [31]. Scores associated with features from clinical history and physical exam and chest X-ray translate to risk of TB and are developed from analysis of diagnostic evaluations. TB—tuberculosis, HIV—human immunodeficiency virus, mWRD—molecular WHO-recommended rapid diagnostic test, CLHIV—children living with HIV, LF-LAM—lateral flow urine lipoarabinomannan assay, CXR—chest X-ray.

A comprehensive review of a large and geographically diverse cohort of children being evaluated for childhood TB commissioned by WHO developed two treatment decision algorithms included in the operational handbook accompanying the 2022 consolidated guidelines on the management of TB in children and adolescents [19]: one for use in settings with CXR and one for use in settings without CXR. (The algorithm for use in settings with CXR is reproduced in Figure 2) Detailed practical guidance on their use and development are included in the operational handbook [31], as well as an accompanying scientific publication (in preparation).

The WHO algorithm first directs the healthcare worker to evaluate for signs that may require urgent management/referral to higher care. The following step stratifies children based on risk of TB-associated mortality to change the threshold for decision making, encouraging faster treatment decision making for children at high risk of disease progression and death. The algorithm then guides the healthcare worker to identify features/combinations of features from the clinical evaluation to inform TB treatment decision making.

Ideally, respiratory specimens (expectorated sputum, sputum obtained by induction, gastric aspirate, nasopharyngeal aspirate, or stool) should be collected for microbiological confirmation, and CXR should be performed whenever possible. CXR is helpful in assisting both TB and alternative diagnoses; it may indicate whether the child has non-severe TB disease that makes them eligible for a shortened (4-month) TB treatment regimen [32]. Lateral flow urine lipoarabinomannan assay (LF-LAM) may also assist diagnosis among children living with HIV. However, TB treatment should not be delayed if the child meets sufficient probability of TB disease criteria (as defined in the algorithm) and CXR, microbiologic testing, or LF-LAM are all not available. Follow-up evaluation is recommended for all children, regardless of whether they were started on TB treatment, to assess for persistence of symptoms and to monitor for adverse drug events among those started on treatment.

WHO’s position to promote the use of data-driven algorithms to inform TB treatment decision making is an important step forward in providing evidence-based pragmatic guidance to scale-up TB diagnosis and treatment access. Additional studies are required to inform the validity and acceptability of incorporating these algorithms into clinical practice.

## 2. Conclusions

Optimizing clinical approaches to TB treatment decision making is important to improve treatment access in TB-endemic settings. The evidence suggests that among children at high risk for TB, a symptom screen differentiates those who should be investigated further for TB disease from those who are unlikely to have TB disease and should receive TPT. For symptomatic children being investigated for TB disease in resource-limited settings, an algorithmic approach may be sufficient to guide TB treatment initiation, even in the absence of imaging or microbiological testing. The urgent need to increase TB detection and treatment access in order to reduce TB-related mortality must be balanced against the consequences of over-diagnosis and unnecessary treatment. Symptom-based clinical approaches provide an opportunity to reduce persistent gaps in TB prevention and treatment.

## Figures and Tables

**Table 1:** Accuracy * of symptom-based screening to exclude tuberculosis disease in child tuberculosis contacts in studies from Indonesia and South Africa.

Reference Standard Used ^	Sensitivity (%)	Specificity (%)	NPV (%)
***Triasih 2015*** All children with culture-confirmed or clinical (at least one well-defined symptom and consensus of two experts on chest X-ray) TB diagnosis at baseline	21/21 (100)	171/248 (69.0)	171/171 (100)
***Kruk 2006, Case definition 1*** All children treated for TB	25/33 (75.8)	168/219 (76.7)	168/176 (95.5)
***Kruk 2006, Case definition 2*** All children with “certain TB” on chest X-ray (as judged by two independent reviewers)	22/27 (81.5)	170/225 (75.6)	170/175 (97.1)
***Kruk 2006, Case definition 3 ^#^*** All children with “certain TB” on chest X-ray, excluding those with asymptomatic hilar adenopathy	22/22 (100)	175/230 (76.1)	175/175 (100)

TB—tuberculosis, NPV—negative predictive value. * This refers to the accuracy of symptom-based screening against the reference standard specified. The symptoms assessed are detailed in the text. ^ Although the reference standards listed are all susceptible to incorporation bias, they all included an independent or objective component, such as a decision by the managing clinicians (not involved in the study) or the impression of independent clinical experts or chest X-ray readers not involved in the management decision. This summarizes the best available data and with full transparency of the reference standard used. ^#^ This case definition demonstrates that the only cases missed by symptom-based screening were children with uncomplicated hilar adenopathy on chest X-ray, which is often asymptomatic and transient following recent primary infection [4].

**Table 2:** Summary of the evidence related to symptom-based screening of child tuberculosis contacts and children living with HIV in high-TB incidence settings.

	Child TB Contacts	Children Living with HIV
Characteristics of screening	If asymptomatic, significant TB disease among child contacts <5 years is unlikely and initiation of TPT is safe	Given somewhat lower sensitivity of symptom-based screening and risk for rapid progression of disease, asymptomatic children need regular, ongoing screening
	At least for those <5 years old, CXR and immunologic tests of infection are not necessary to determine eligibility for TPT if a child is asymptomatic	Symptom screening alone is likely effective for determining which children can initiate TPT
Limitations in evidence	Lack of a point-of-care test for infection and disease susceptibility that reliably determines effective and efficient use of TPT	TB exposure risk, especially undocumented exposure outside of the household, is highly dependent on the setting
	Safety of symptom screening alone to determine eligibility for TPT requires more study, especially in children ≥5 years of age	Accuracy of screening may differ widely if on ART and depending upon degree of immunosuppression
	Is CXR required in asymptomatic child contacts to detect/exclude active TB?	Optimal frequency of screening, particularly for those on ART and TPT, is not well established
	Need for further evidence of the additional benefits/risks/operational challenges of including a positive test for infection to determine eligibility for TPT	

TB—tuberculosis, HIV—human immunodeficiency virus, CXR—chest X-ray, TPT—TB preventive treatment.

**Table 3:** Clinical trade-offs in deciding to initiate tuberculosis treatment in a child.

	Implications	
Decision	Positive	Negative
Initiate treatment early	Reduce risk of TB-associated morbidity/mortality due to rapid TB disease progression in highly vulnerable children	Potential to miss alternate (non-TB) diagnoses that may carry their own morbidity/mortality risk
	Evidence from clinical history and recent TB exposure may be sufficient to begin TB treatment	Adverse drug events associated with unnecessary TB treatment if true diagnosis is not TB (though TB treatment is generally well-tolerated)
		Inconvenience and cost of unnecessary TB treatment
		Potential to undermine patient trust in the healthcare system if true diagnosis is not TB
Withhold/delay treatment	Potential to increase specificity by follow-up for persistence of symptoms in a child with no danger signs	Risk of TB-associated morbidity/mortality due to progression of TB disease if lost to follow-up (progression of disease is possible, but less likely if follow-up is within 1–2 weeks)
	Opportunity to pursue alternate (non-TB) diagnosis and assess response to alternate treatment	
	Time to obtain results from diagnostic imaging and microbiological or other tests	

TB—tuberculosis.
